# Age-Related Characteristics of Resting-State Electroencephalographic Signals and the Corresponding Analytic Approaches: A Review

**DOI:** 10.3390/bioengineering11050418

**Published:** 2024-04-24

**Authors:** Jae-Hwan Kang, Jang-Han Bae, Young-Ju Jeon

**Affiliations:** 1 Digital Health Research Division, Korea Institute of Oriental Medicine, Daejeon 34054, Republic of Korea; jh.kang@kiom.re.kr (J.-H.K.); fcbest11@kiom.re.kr (J.-H.B.); 2 Aging Convergence Research Center, Korea Research Institute of Bioscience and Biotechnology, Daejeon 34141, Republic of Korea

**Keywords:** age-related changes, resting-state EEG signals, analytic methods, age-related AI models

## Abstract

The study of the effects of aging on neural activity in the human brain has attracted considerable attention in neurophysiological, neuropsychiatric, and neurocognitive research, as it is directly linked to an understanding of the neural mechanisms underlying the disruption of the brain structures and functions that lead to age-related pathological disorders. Electroencephalographic (EEG) signals recorded during resting-state conditions have been widely used because of the significant advantage of non-invasive signal acquisition with higher temporal resolution. These advantages include the capability of a variety of linear and nonlinear signal analyses and state-of-the-art machine-learning and deep-learning techniques. Advances in artificial intelligence (AI) can not only reveal the neural mechanisms underlying aging but also enable the assessment of brain age reliably by means of the age-related characteristics of EEG signals. This paper reviews the literature on the age-related features, available analytic methods, large-scale resting-state EEG databases, interpretations of the resulting findings, and recent advances in age-related AI models.

## 1. Introduction

Aging affects the entire human brain, in which the anatomical and functional alternations in the cellular structures extend to massive changes in neuronal morphology and neural plasticity [1,2,3]. In particular, because age-related changes in the neural system in adults over the age of 60 are inevitably associated with a risk of neurodegenerative diseases with behavioral and cognitive impairments [4,5], recent studies have attempted to understand how aging affects the brain and what kinds of neural characteristics manifest, in addition to investigating how brain age can be quantitatively assessed using artificial intelligence (AI) in combination with the corresponding age-related neural evidence [6,7,8,9]. In brief, aging is known to mediate the morphology of neurons and synaptic plasticity differentially in the hippocampus and cerebral cortex [10,11]. A decline in neurotransmitters, such as dopamine, serotonin, and neurosteroids (testosterone in men and estrogen in women), in the hippocampal formation leads to the degradation of neural mechanisms across associated regions and may finally cause senile neuropathological disorders, such as dementia, Alzheimer’s disease and Parkinson’s disease [4,12,13]. Structural or functional neuroimaging studies on the effects of aging on the brain have reported that aging decreases the volume and integrity of both gray and white matter in the cerebral cortex, despite different patterns of declines in each matter type [11,14]. Due to the different vulnerabilities to aging in the subregions of the brain, volumetric changes in the medial temporal lobe (MTL) and prefrontal cortex (PFC) are more significant rather than those in the parietal and occipital regions [2,11,15]. Neuroscience studies on human aging have employed neural activities that can be recorded noninvasively, such as electromagnetic changes and hemodynamic fluctuations, to elucidate the underlying neural mechanisms of aging. In particular, scalp electroencephalography (EEG) has been widely used to estimate or predict brain age because it allows the easy acquisition of neural signals in a more comfortable and natural environment than other techniques, such as functional magnetic resonance imaging (fMRI), diffusion tensor imaging (DTI), magnetoencephalography (MEG), and positron emission tomography (PET) [6,16,17,18,19].

In terms of digital signal processing, EEG has excellent temporal resolution but relatively low spatial resolution without additional source-localization methods compared with blood oxygen level-dependent (BOLD) image-based signal techniques [20]. These advantages of EEG allow for various types of time-series signal processing and analytical approaches based on both linear and nonlinear models [20,21,22]. Linear spectral analysis, which explains a single EEG signal as a combination of several different oscillatory components, uses physical properties, such as the amplitude or phase of each oscillatory component [23,24]. Nonlinear analysis attempts to interpret time-series signals in terms of nonlinear dynamic properties, such as regularity, complexity, and predictability [21,25]. Various brain connectivities based on the corresponding mathematical principles have been proposed to identify the significant evidence of functional (unidirectional) or effective (bidirectional) connectivity in the brain using graph-theory-based network analysis [26,27]. Accordingly, many neuroscience and engineering studies on aging have focused on the characteristics of EEG signals to identify their relationship with aging or create mathematical models for age classification or prediction [6,7,8,9]. This review intensively focuses on normal aging EEG signals recorded during the awake resting state with eyes closed (REC) or eyes opened (REO) without any cognitive task engagements and then extensively surveys the literature on how aging affects the intrinsic characteristics of EEG signals and the relevant methodological approaches. Compared with the task-relevant condition, in which individuals perform a specific cognitive task or are exposed to an external stimulus, neural activity in a task-irrelevant resting state has been reported to be strongly linked to the intrinsic or innate properties of an individual’s brain state [28,29,30]. Therefore, many EEG and fMRI studies on the prediction of human aging have mainly focused on neural activities recorded during the resting state [27,31,32,33]. By examining rsEEG signals, they attempt to determine how aging changes the EEG signal, how this can be expressed through certain EEG indices, and how this relates to brain developmental processes throughout the lifespan, including growth, maturation, and aging [30,34].

The remainder of this paper is organized as follows. First, we present several large-scale EEG databases that have been used in previous age-prediction studies. Second, we discuss age-related EEG measures that can be extracted from a single channel using traditional analytical approaches, such as linear spectral analysis or nonlinear dynamics models. Third, we describe the age-dependent properties of the spatial components or measures of brain connectivity extracted from multiple EEG channels, including findings on the influence of aging on the properties of EEG microstates or functional connectivity in combination with network analysis. Finally, we introduce a state-of-the-art EEG processing in the manifold space (i.e., the Riemannian manifold), along with several ML- and DL-based regression models or classifiers to predict aging.

## 2. Large-Scale EEG Database Related to Aging

Previous EEG studies on developmental stages of the brain or aging in healthy individuals can be roughly divided into two types of EEG acquisition conditions in which EEG signals were recorded while performing goal-specific tasks or during a resting state with eyes closed (REC) or opened (REO) [35,36,37]. In goal-specific tasks, the recorded EEG signals are analyzed on the basis of the task corresponding to the experimental paradigm. Most EEG signals are recorded during a specific cognitive process, such as working memory, attention, and emotion; therefore, the corresponding behavioral results are commonly included. In contrast to task-relevant EEG signals, which are linked to cognitive performance as measured by behavioral data, task-irrelevant rsEEG signals have more intrinsic and biological properties that are independent of the presence of external stimuli or the performance of specific cognitive tasks [27,38]. For these reasons, previous EEG studies on aging have aimed at creating rsEEG databases to identify significant differences or unique classifications between two or three specific age groups, but also at revealing specific EEG relationships across the human lifespan. Unfortunately, most of these databases are not publicly available and include only a small number of participants, divided into two or three age groups, such as young and older groups, or young, middle-aged, and older groups with a total of fewer than 100 participants. Some brain research consortia have published individual, large-scale rsEEG databases with more than 100 participants, reporting resting-state multichannel EEG signals from infancy to older age and containing additional information on individual physical and personality traits [39,40]. In this article, we limit ourselves to large-scale and open-access rsEEG databases that fulfill the criteria of including at least 100 participants and as many age groups as possible.

### 2.1. Temple University Hospital Abnormal EEG Corpus

The Temple University Hospital (TUH), Philadelphia, USA, has released a series of large-scale EEG databases consisting of several EEG records and information annotated by clinicians. As of September 2023, seven major EEG databases have been released by the TUH, including the TUH EEG corpus (TUEG), TUH abnormal EEG corpus (TUAB), TUH EEG artifact corpus (TUAR), TUH EEG epilepsy corpus (TUEP), TUH EEG events corpus (TUEV), TUH EEG seizure corpus (TUSZ), and TUH EEG slowing corpus (TUSL) [41,42]. Among these, the TUAB has been widely used in EEG studies on aging [6,7,8,43]. This database is consistently updated, and the 2023 version is 2.0.0. This database has also been adopted by the EEG studies on general properties of multiple EEG signals because it consists of two types of EEG recordings, normal and abnormal EEG signals, based on whether the EEG signals comprise or do not comprise five unique characteristics, namely reactivity, alpha rhythm, mu rhythm, beta activity, and theta activity [44]. The TUAB comprises EEG records constituted of multiple EEG signals with 20–30 channels at a 250 Hz sampling rate during at least 15 min of a resting state. A total of 2993 datasets, consisting of 1521 normal and 1472 abnormal EEG signals, are stored in two different folders for evaluation and training datasets in a ratio of 1:9. All individual EEG records correspond to the individual information annotated in the text for biological information, such as age, sex, and clinical information, such as specific brain disorders diagnosed by physicians. Several studies on ML- or DL-based models have used this database to predict the biological age of individuals [6,7,8,9,43].

### 2.2. Leipzig Study for Mind–Body–Emotion Interactions Database

The Leipzig study for mind–body–emotion interactions (LEMON) [39] is a large-scale multimodal body dataset, released by the Max Planck Institute for Human Cognitive and Brain Sciences in Leipzig, Germany, in 2018. The LEMON database consists of EEG and T1/T2 magnetic resonance imaging (MRI) image data, as well as behavioral data from questionnaires to assess individuals’ cognitive traits. All data were obtained during four individual experimental rounds between 2013 and 2015. To collect the rsEEG recordings, 227 participants, including 154 young participants in the age group 20–25 years and 74 older participants aged 59–70 years, participated in the EEG recording sessions. These sessions consisted of 16 individual experimental blocks in which the 62-channel EEG signals were recorded for 1 min by two types of REO or REC, alternating them so that there were eight REO blocks and eight REC blocks. Several age-related EEG studies have used the LEMON database for comparison purposes. One study compared the predictive power of biological age using five different approaches [6]. Using this database, aging has been reported to influence distinct EEG spatial patterns, such as EEG microstates [45], signal variability in beta rhythms [46], and long-range temporal correlations in alpha rhythms [47].

### 2.3. Cuban Human Brain Mapping Project Database

In 2021, the Cuban Neuroscience Center (CNEURO) published a multimodal neuroimaging and cognitive dataset called the “Cuban human brain mapping project” (CHBMP), which was collected between 2004 and 2008. This dataset consists of high-density (64–120 channels) rsEEG signals, T1 MRI and diffusion-weighted (DWI) images, various behavioral outcomes from psychological testing, and demographic information from 282 young and healthy middle-aged participants. The study population comprised 87 women and 195 men aged 18–68 years [48,49]. Another project on pediatric and older age groups is currently in progress. EEG signals in the CHBMP dataset were recorded for at least half an hour according to the following six protocols: (1) baseline: REC for 10 min, (2) reactivity: consecutive REO and REC with an interval of 12 s for 5 min, (3)–(5) hyperventilation (HPV): inhaling air through the nose thrice and breathing deeply for 1 min, and (6) recovery: recovery after HPV inspiration for about 2 min. In order to extend age-related EEG studies, this project, in collaboration with international research groups, investigated descriptive parameters for aging in EEG signals [50]. This research consortium developed a reliable algorithm for predicting biological age by transforming the descriptive parameters of the EEG, which are based on the cross-spectral density of several EEG signals, into the Riemannian manifold dimension [50]. As described in the introduction to the LEMON database, Engemann et al. used the CHBMP, LEMON, and TUAB databases to assess the performance of five representative algorithms for predicting brain age [6].

### 2.4. Tulsa 1000 Database

The Laureate Institute in Tulsa, USA has released a large-scale multimodal database that includes fMRI images and concurrent 31-channel EEG signals of 1000 participants [40]. Considering dimensional psychopathology beyond traditional psychiatric diagnoses, the participants consisted of healthy controls and treatment-seeking individuals with mood, anxiety, substance use, and eating disorders. In addition, insightful behavioral results were obtained from various questionnaires for the domains of positive and negative valence, cognition, and arousal [40]. However, unlike TUAB, LEMON, and CHBMP, this database is not open access. In addition, most of the studies used in this database are from the Laureate Institute. In one study, an automated pipeline for artifact reduction of EEG signals in this database was presented, since the EEG signals in this database were contaminated with MRI gradients and ballistocardiogram artifacts due to simultaneous EEG recording in magnetic resonance scanners [51]. Using the EEG signals in this database, Al Zoubi et al. indicated significant differences in the temporal dynamics of EEG microstates between healthy controls and individuals with mood and anxiety disorders [52]. They also investigated the performance of classification or regression of several ML approaches to predict brain age [53].

### 2.5. ISB-NormDB (QEEG Normative Database)

A commercial healthcare company in Republic of Korea, iMediSync Inc. (Seoul, Republic of Korea)., provided a sex- and age-differentiated normative EEG database called the ISB-Norm database [54]. This database is not publicly accessible. A total of 1289 participants, including 553 men and 736 women aged 4.5–81 years, were recruited for this database. The 19-channel EEG signals, digitized at a sampling rate of 250 Hz, were recorded for 4 min REO and REC, each. Based on the age groups of the participants, different questionnaires were administered to assess individual cognitive, emotional, and behavioral characteristics. Using this database, one study showed that several spectral powers of the delta, theta, alpha, beta, and gamma rhythms were modulated by the ages of the participants and correlated strongly with those of the commercial EEG database, such as qEEG-Pro [54]. A specific algorithm was developed that achieved 92.3% accuracy in discriminating between 116 potentially depressed participants and 80 healthy controls using four representative EEG features as absolute or relative spectral powers [55].

### 2.6. Other Databases

Several other databases are available for commercial services or research on age-related EEG changes. However, most of these studies fall outside the scope of our review, as they do not fulfill the criteria of including more than 100 participants of all ages. Nonetheless, some noteworthy databases relevant to EEG research on aging are briefly described below. Several valuable large-scale databases intended for noncommercial research purposes did not focus on healthy participants, but on pediatric participants with a neurodevelopmental stage from infancy to early adulthood or on pathological patients targeting different types of psychiatric disorders. The Healthy Brain Network (HBN) project, for example, recruited about 10,000 participants aged 5–21 years and created open-access databases of fMRI and EEG signals and various behavioral questionnaires [56]. In a recent study, this database was used to identify changes in the characteristics of the periodic and aperiodic components of brainwaves at different stages of brain development [57]. Another project recruited more than 10,000 participants aged 40–79 years and established the LIFE Adult study database. This database contains extensive physiological information and comprises 20 min rsEEG datasets from participants aged ≥ 60 years [58]. Although it is a nonpublic database, recent research utilizing it has shed light on the relationship between two major age-related alterations, including individual alpha frequency (IAF) decline and flatter 1/f spectral slope, and cognitive performance [59]. To support the development of biomarkers and methods to address psychiatric dysfunctions, Two Decades-Brainclinces (TDBrain) Research has released an open-access EEG dataset called the “TDBrain database”. This database contains 2 min REO and REC EEG recordings from 1274 psychiatric patients aged 5–89 years, including patients with major depressive disorder (MDD; N = 426), attention deficit hyperactivity disorder (ADHD; N = 271), subjective memory complaints (SMC; N = 119), and obsessive–compulsive disorder (OCD; N = 75) [60]. In contrast to noncommercial research databases, several private EEG databases are only available for commercial services. These databases, such as qEEG-Pro, BrainDx, NeuroGuide, and HBimed, offer commercial services that involve analyzing signals from input brainwave data using proprietary technologies and databases and providing evaluations as a result.

## 3. Age-Related Spectral Measures

Several critical EEG characteristics or features related to the aging of the brain have been proposed in the past [35,61,62,63,64,65]. Spectral analysis is the most commonly used technique in EEG analysis methods to decompose a single EEG signal into a set of different spectral components, generally including delta (1–4 Hz), theta (4–8 Hz), alpha (8–12 Hz), beta (12–30 Hz), and gamma (above 30 Hz) rhythms. The method is also employed to evaluate the spectral measures of each component by using the spectral power or phase information within the corresponding frequency band [24,66]. In contrast to these oscillatory rhythms, another critical component of neural activity is defined as an aperiodic component or scale-free 1/f noise [67,68]. Previous studies have revealed the critical role of 1/f noise in neural mechanisms underlying cognitive and neural processing [67,68,69,70,71]. In fact, the aperiodic component is not only strong interference in the feature extraction of the periodic component [72,73,74,75,76] but is also an important factor in the assessment of human aging [30,68,77,78]. For this reason, some studies on aging have attempted to develop new algorithms, in which EEG signals are first divided into aperiodic (1/f noise) and period components [72,73,74]. Next, the aperiodic component is eliminated to obtain the spectral power or is converted into independent features (slope and offset) to characterize the power spectrum density (PSD) of 1/f noise [72,73,74].

In this session, we will describe the age-dependent spectral measures proposed in previous studies. However, the corresponding results remain controversial. Various spectral methods to calculate spectral power and different groups related to the participants’ ages make it difficult to interpret the findings directly. For example, some studies used absolute spectral power, whereas others used relative spectral power. Depending on the type of power used, absolute, relative, or aperiodic-adjusted spectral powers, the resulting spectral characteristics of aging differ significantly. Moreover, distinct developmental procedures in the brain have to be considered based on the participants’ ages, which are roughly grouped into three perspectives: developmental cognitive neuroscience from childhood to early adulthood (<25 years old), degenerative neuropsychiatric disease in older adults (>70 years old), and neurophysiological changes throughout the human lifespan [30,32,79]. Not all spectral measures exhibit significant and linear age-related changes statistically. However, some studies jumped to general conclusions based on specific patterns within a narrow age range. For these reasons, we tried to consider not only the method of power calculation but also the number and age of each group of participants. The age-related spectral measures are described under the following subheadings: (1) power slope, (2) alpha rhythm: peak frequency, (3) alpha rhythm: power, (4) alpha rhythm: reactivity, (5) beta power, and (6) other spectral powers.

### 3.1. Power Slope (1/f Noise)

Aperiodic components are generally quantified as a measure of the slope or offset of the PSD of EEG signals. This increase in power slope in the PSD is determined by the balance between excitatory and inhibitory inputs, which consist of fast glutamatergic and slow GABAergic synaptic connections, respectively [80,81]. This balance in synapses provides the basis for the maintenance of neural homeostasis and the formation of neural oscillations in terms of information transmission and computation in the brain network [70]. One of the most consistent findings in age-related EEG measurements is the power slope of the PSD. The power slope flattens with age because excitability and neural noise increase, leading to asynchronous spiking activity. In addition, population-level synchrony has been reported to decrease in neural networks [59,68,69,77]. Since aperiodic components in EEG rhythms are highly related to aging but also affect the calculation of periodic power, several studies have proposed a bundle of efficient algorithms to obtain the spectral powers more purely by separating aperiodic components and performing a further statistical analysis. Donoghue et al. proposed a new algorithm called fitting oscillations and one over f (FOOOF). In FOOOF, a neural signal is first separated into aperiodic and periodic components [72]. Then the aperiodic component, which corresponds to the 1/f properties of the neural signal, is eliminated. Finally, the spectral power is calculated from the pure periodic component only [72]. Whitten et al. proposed a new algorithm called the better oscillation detection (BOSC) method [73]. This algorithm first identifies the nonrhythm activity components as background by fitting the empirically observed power spectrum in the neural signal. Then it calculates the spectral power from the remaining components that significantly deviated from the spectral characteristics of the background [73]. Wen and Liu proposed irregular resampling autospectral analysis (IRASA) to robustly separate the fractal (aperiodic) component [73]. It was developed to extract the fractal power by computing the auto-power spectra of a bundle of resampled time-series signals modified from the original signals in terms of Hurst exponent and Fourier transform [74].

Recent EEG studies have consistently shown the obvious effect of aging on aperiodic components (Table 1). Donoghue et al. examined not only the differences in the periodic components of alpha oscillations, such as IAF and power but also the aperiodic components of PSD, such as offset and exponents (same as slope), between younger (20–30 years, N = 17) and older (60–70 years, N = 14) adults. By applying the FOOOF algorithm, they found not only two age-dependent periodic measures (older adults had slower alpha center frequencies and lower aperiodic-adjusted alpha power) but also two age-dependent aperiodic measures (older adults had lower aperiodic offset and flatter aperiodic exponents) [72]. Pathania et al. examined the 2 min REC signals obtained from two different age groups: healthy young adults (<35 years, N = 22) and healthy older adults (>59 years, N = 24). Using the FOOOF algorithm, they found that older adults had significantly lower (flatter) exponents of the aperiodic components in the frontal, central, and parietal regions than young adults [82]. Cesnaite et al. examined the REC signals from participants (40–79 years; N = 1703) in a large physiological dataset, named the LIFE-Adult dataset, and reported two significant negative relationships with aging by using source reconstruction: (1) IAF in the left temporal lobe and (2) 1/f slope in the right frontal lobe. The results of the abovementioned three recent studies have shown that the PSD in rsEEG signals becomes flatter. That is, more noise was noted with increasing age throughout the lifespan [59]. Unlike some spectral components, which present a U-shaped or nonlinear relationship across the lifespan, these properties of the aperiodic components have a relatively linear trend during the developmental period. Recent studies have focused on the significant changes in the aperiodic component of rsEEG signals during the neurodevelopmental period from childhood to adolescence, when there is significant growth and fundamental neural reorganization throughout the cortex [57,78,83]. Cellier et al. analyzed REO signals from 96 participants aged 3–24 years [78]. Tröndle et al. used a large-scale pediatric database (5–22 years, N = 2529) and independent validation (6–22 years, N = 369) [57]. Hill et al. recruited participants from early-to-middle childhood ranging between 4 and 12 years (9.41 ± 1.95 years, N = 139) [83]. Regardless of the different age groups and sample sizes in the participant groups, these studies consistently found that both the aperiodic slope and the offset (intercept) decreased, but the peak frequency in the alpha rhythm increased with age.

### 3.2. Alpha Rhythm

The alpha rhythm was recognized earlier than the other brain rhythms [84]. It generally ranges between 8 and 13 Hz, the most dominant rhythm in human EEG signals [63,85]. In the past, the alpha rhythm was regarded as the representation of the idling state in the brain due to its enhancement in task-irrelevant conditions as opposed to other rhythms. However, the alpha rhythm has distinctive characteristics compared to the other rhythms, but it is also highly associated with a variety of task-relevant tasks [86], such as working memory [87], attention [88], facial preference [89], perceptual learning [90], and top–down inhibitory process [91]. The alpha rhythm has been reported to be closely linked to the resting state or default-mode network in the brain. For example, a concurrent EEG-fMRI study investigated the relationship between the temporal dynamics of neural activity from EEG signals and the spatial distribution of brain areas from resting-state fMRI images [38]. It showed that alpha activity in EEG signals correlated positively with BOLD activity in the resting-state network. However, it negatively correlated with those in the dorsal attention network. The enhancement of alpha activity seen in the visual cortex and posterior region during REC is strongly suppressed by the presence of visual stimuli [38]. This alpha suppression is known to facilitate the transmission of sensorimotor information and memory processes [87,92,93]. In addition, the alpha rhythm is known to play an important role in the functional feedback in the thalamocortical system by propagating from the higher- to lower-order cortex and from the cortex to the thalamus [94,95].

Compared with other rhythms, the alpha rhythm is the most prominent rhythm influenced by human aging [18,93,96,97]. This alpha modulation due to aging has been associated with physiological changes in white- or gray-matter volume [32,98] and cognitive performance in working memory [93]. As we focus more on the age-related characteristics of the alpha rhythm at rest, we have selected three important alpha characteristics: individual alpha frequency, alpha reactivity, and alpha power (Table 2).

#### 3.2.1. Alpha Peak Frequency

One of the well-known EEG characteristics of aging is that EEG rhythms, especially the theta and alpha rhythms, become slower with increasing age [23,63,64,99]. Since a shift toward slower frequencies is reflected in the IAF or center frequency within the alpha rhythm with increasing age, the older group was generally reported to be slower than the younger group [64,100]. A comparison of alpha peak frequency between young and old groups in two studies has revealed that frequencies in the older group were significantly lower than those in the younger group [23,99]. The study by Scally et al. recruited a total of 69 participants belonging to two age groups, 37 in the young (20.3 ± 2.1 years) and 32 in the older (69.8 ± 4.9 years) groups. In addition to the slower alpha peak frequency with aging, the authors reported that the old group had lower alpha power and weaker global connectivity than the young group [99]. The study by Stacey et al. also recruited 75 participants from two age groups, 31 young (24.0 ± 4.5 years) and 44 older (71.5 ± 6.5 years), and was mainly concerned with the fact that the older group had a lower alpha peak frequency but a higher beta power compared to the young group [23]. However, a slower alpha peak frequency with age was not observed across the lifespan. Several developmental EEG studies, focusing on individuals under the age of 25 years, reported a relationship between alpha peak frequency and increasing age [57,78,83]. A more recent study on the characteristics of alpha oscillations in childhood and adolescence reported that the alpha peak frequency increased linearly with age, ranging 5–22 years of age (N = 2529) [57].

With regard to alpha spatiality, the effects of aging on the alpha rhythm have been reported to be mainly observed in the posterior region. By examining peak alpha frequency in 60 participants aged 20–81 years, Knyazeva et al. identified two components of the alpha rhythm that were distributed in the posterior region [101,102]. These included higher frequency components (peak: 9.9 ± 0.8 Hz) from the occipito-parietal regions and lower frequency components (peak: 9.0 ± 0.9 Hz) from the occipito-temporal cortices [101]. The peaks of the higher frequency components, which were more distributed over the posterior regions, decreased more markedly with increasing age. However, the low-frequency component was relatively insensitive to age [102]. A study with 96 participants aged 3–24 years showed that the alpha peak frequency in the posterior region during REO shows a linear increasing trend with age [78]. Furthermore, the dominant frequency extracted from the parietal-midline areas was mainly in the theta band (4–8 Hz) in infants but shifted to the alpha band (8–12 Hz) in adolescents [78]. Another study, including 139 participants aged 4–12 years, also reported similar findings that the alpha center frequency of the posterior area from both REO and REC increased with age [83]. In addition to these findings, several studies covering the entire human lifespan have reported that alpha peak frequency, particularly in the posterior region, increases linearly at around 20 years of age and begins to decline at around 40–50 years of age [96,103]. To summarize, aging strongly modulates the alpha rhythm, which is distributed over the posterior region, compared to other regions. The peak alpha rhythm increases linearly with age during the developmental stages of the brain but gradually decreases with the process of brain degeneration. A decrease in peak alpha frequency leads to a shift to slower frequencies in all EEG signals with increasing age. In particular, a more obvious slowing in the posterior regions leads to a shift in the brain’s neural network from the posterior to the anterior region with age [34,104]. This is described in detail in Section 5.1.

#### 3.2.2. Alpha Reactivity

Another important feature associated with the aging of the alpha rhythm is alpha reactivity. Particularly in the posterior region, alpha reactivity or suppression refers to a marked decrease in alpha activity in response to the presence of a visual stimulus or to the performance of cognitive tasks that require cognitive attention or mental effort [97]. Alpha suppression is thought to reflect the active processing of sensory information, as it facilitates task-relevant areas and allows other neural frequencies to become more prominent. Apart from different calculation methods, alpha reactivity is generally defined as the difference in alpha power between two resting-state conditions, in which the participant’s eyes are either closed or open. Human aging has been reported to strongly influence the characteristics of alpha activity [18,31]. Several studies on age-related alpha reactivity have argued that this relationship is significant in the older group but not in the younger group [36,105,106,107]. A study with 63 participants aged 30–80 years showed that both types of alpha reactivity, which are calculated by the difference (ECALM-EOALM) or ratio (ECALM/EOALM) of alpha amplitude between the eyes-closed state (ECALM) and eyes-open state (EOALM), correlated negatively with aging [35]. Another study that recruited old (65.1 ± 1.18 years, N = 32) and young (22.1 ± 0.38 years, N = 33) participants also indicated lower alpha reactivity in the old group in comparison to the young group [107]. Indeed, this negative correlation between alpha reactivity and aging is more apparent in older groups than in younger groups [36,105,106,107]. An EEG study on aging investigated the difference in alpha reactivity, as the ratio of REC and REO in alpha power, between two subgroups (young and old groups) of healthy adults (52.9 ± 19.0 years, N = 54). The authors observed a nonlinear relationship between alpha reactivity and age in the temporo-occipital regions. Furthermore, a significant negative correlation with age was only observed in the old group [105]. Another EEG study examined the difference in alpha reactivity between older adult participants (69.5 ± 10.2 years, N = 39) and young controls (29.9 ± 10.4 years, N = 21) and reported a significant negative correlation with aging only in the older adults group [106]. However, another study pointed out that the differences in performance between REC and REO are not limited to the alpha rhythm in the posterior region but can also be observed in other bands, such as the delta and beta rhythms in noncentral regions and the theta rhythms in the posterior region [37].

**Table 2:** Review of age-related alpha measures of resting-state EEG signals.

Measures	Study	Main Results	Subjects (Age Range (yr), Numbers (N))	Eyes Condition
Alpha frequency	Cellier et al. [78]	peak frequency: young < old	adolescence (3–24 yr, N = 96)	REO
	Hill et al. [83]	center frequency: young < old	early–to–middle childhood (4–12 yr, N = 139)	REO REC
	Tröndle et al. [57]	peak frequency: young < old	adolescence (HBN project) (5–22 yr, N = 2529)	REO REC
	Stacey et al. [23]	peak frequency: young > old	young (18–30 yr, N = 31) old (61–90 yr, N = 44)	REO REC
	Scally et al. [99]	IAPF: young > old	young (20.3 ± 2.1 yr, N = 37) old (69.8 ± 4.9 yr, N = 32)	REC
	Donoghue et al. [72]	center frequency: young > old	young (20–30 yr, N = 17) old (60–70 yr, N = 14)	REO
	Aurlien et al. [103]	peak frequency: increase until 20 yr	0–100 yr (N = 4651)	REC
	Chiang et al. [96]	peak frequency: younger > older	6–86 yr (N = 1498)	REC
	Cesnaite et al. [59]	IAPF: decrease	LIFE–Adult DB (40–79 yr, N = 1703)	REC
Alpha reactivity	Duffy et al. [35]	decrease	30–80 yr (N = 63)	REO REC
	Könönen et al. [105]	decrease	53 ± 19 yr (N = 54)	REO REC
	Marciani et al. [106]	decrease	young (29.9 ± 10.4 yr, N = 21) old (60.6 ± 5.4 yr, N = 20) older (78.7 ± 7.3 yr, N = 19)	REC
	Gaal [36]	decrease	young (21.5 ± 22 yr, N = 23) old (66.9 ± 3.6 yr, N = 25)	REO REC
Alpha power	Tröndle et al. [57]	absolute power: decrease relative, adjusted power: increase	adolescence (HBN project) (5–22 yr, N = 2529)	REO REC
	Babiloni et al. [108]	power: young > old	young (18–50 yr, N = 108) old (51–85 yr, N = 107)	REC
	Scally et al. [99]	absolute power: young > old	young (20.30 ± 2.06 yr, N = 37) old (69.75 ± 4.91 yr, N = 32)	REC
	Knyazeva et al. [102]	higher alpha rhythm (more distributed)	45–81 yr (N = 32)	REO REC
	Donoghue et al. [72]	aperiodic adjusted-power: young > old	young (20–30 yr, N = 17) old (60–70 yr, N = 14)	REO

#### 3.2.3. Alpha Power

Compared to the two age-dependent properties of the alpha rhythm, peak frequency and reactivity, the effect of aging on alpha power is more controversial, as it depends on different methods of calculating alpha power. Alpha power is usually calculated as the mean of absolute or relative powers and is often normalized by a logarithm or other transformations. To obtain a more reliable measure of individual oscillations, some analytical methods for calculating EEG spectral power have evaluated the individual power of the oscillatory rhythm after the elimination of the aperiodic component by additional signal processing. Depending on the power calculations, the resulting correlations with aging are inconsistent [93]. Similar to alpha peak frequency and alpha reactivity, the relationship between alpha power and aging is not linear across the lifespan; therefore, the ages of the participants’ groups must be considered. On the individual spectral power of EEG rhythms associated with aging, the alpha rhythm highly reflects the processes of the physiological development of the brain, such as growth, maturation, and decline [64,78,102]. Alpha power in adolescent and middle-aged groups has been reported to be lower than that in infant and early young groups [57,109,110]; however, it is higher than that of the old group [108]. In a concise review of alpha and theta oscillations, Klimesch succinctly stated that upper alpha power increases from early childhood to adulthood, but gradually decreases later in life [93]. Similarly, several studies have addressed the power or magnitude of the alpha rhythm, which is mainly distributed in the posterior regions and decreases with physiological aging [18,64,72,97,108]. Babiloni et al. indicated that alpha power in the occipital areas, which is spatially specified by low-resolution brain electromagnetic tomography (LORETA), linearly decreased with age in a cohort of young (18–50 years, N = 108) and older (51–85 years, N = 107) participants [108]. Donoghue et al. compared the difference in alpha power between younger and older adults in conditions with two types of alpha powers calculated using the FOOOF algorithm. They pointed out that the inclusion of age-related aperiodic changes exaggerated the effect of aging on the magnitude of alpha power [72]. Several EEG studies have consistently shown a linear increase in relative or aperiodic-adjusted alpha powers with increasing age from 5–22 [57], 8–12 [109], and 5–12 years [111].

### 3.3. Beta Power

The beta rhythm is an oscillatory rhythm that generally ranges between 12 and 30 Hz, with an onset at approximately 12–13 Hz. The “sensorimotor rhythm” [84] is closely associated with motor activity in the body. It is widely adopted in brain–computer interface (BCI) research, where it enables the control of machines through imagery or voluntary modulation of movement [112]. In addition, the beta rhythm is considered a rhythm that indicates the state of equilibrium of neural activity in the brain, as it is often observed in stable postures, while it disappears during movement [113]. For these reasons, this rhythm is closely associated with processes involving executive control of movement [113], such as in Parkinson’s disease [114,115], and serves as a significant marker for arousal processing, as observed in conditions such as ADHD [116,117]. In studies analyzing BOLD and EEG signals, beta power has been reported to have strong associations with a few different brain networks, including positive correlations with a resting state and self-referential brain network activity and negative correlations with dorsal attention network activity [38]. Similarly, in a study by Laufs et al., while not visible in the 13–16 Hz range, beta rhythm activity in the 17–23 Hz range was found to exhibit significant characteristics of the default mode network, which aligned with the distribution of task-independent deactivations [118].

Several rsEEG studies have also examined the effects of aging on beta rhythms (Table 3). Although there are some contradictory findings, the consensus is that beta power tends to increase with age. For example, using a young group (average age approx. 30 years, N = 21) as a control group, the researchers examined two older groups: one aged 50–69 years (old, N = 20) and the other aged 72–86 years (older, N = 19) by measuring the rsEEG of the central and occipital regions [106]. They calculated and analyzed the relative power in different frequency bands, finding a significant difference was observed in the beta rhythm in the range of 13–19.5 Hz and no significant differences between the young and old groups in other frequency bands. In the older group (50–86 years, N = 39), the beta power showed a linear correlation with age and a linear increase with a correlation coefficient of about 0.55 [106]. Stacey et al. analyzed and compared the REO and REC EEG between a young group aged 18–30 years (N = 31) and an older group aged 61–90 years (N = 44). They reported a statistically significant increase in the occipital beta power and a decrease in the IAF of the global area in the older group [23]. Similarly, a study targeting 40 healthy participants aged 6–65 also found a linear increase in the relative beta power in the frontocentral area with advancing age [119]. Using the BOSC method, one study investigated the relationship between valid spectral powers in relation to age and found that only beta power, which was not seen in other bands in the ≥12 Hz range, showed an increase in the older group (60–74 years, N = 12) compared to the younger group (18–25 years, N = 16) [100]. In contrast to these results, however, a study comparing a relatively small group of 25 people, with an average age of 23 and 70 years in the young and older groups, respectively, found a significant reduction in absolute beta power in the midline and occipital areas in the older group [62]. Interestingly, a recent study focused on the variability of the beta rhythm and not on performance. The authors analyzed the rsEEG signals in LEMONDB and reported that the variability of the beta rhythm amplitude across temporal regions increased with aging [46].

### 3.4. Other Spectral Powers—Delta, Theta, and Gamma Rhythms

Compared to the characteristics of the alpha and beta rhythms described above, fewer studies have found statistically significant correlations between age and the spectral measures of the delta, theta, and gamma rhythms. Regarding the delta rhythm, one EEG study, which analyzed the REC signal by the LORETA technique, reported that the young group (18–50 years, N = 108) showed a higher delta power in the occipital regions compared to the older group (51–85 years, N = 107) [108]. Another study on the age-related relationship between EEG spectral parameters and gray- and white-matter volume reported a significant decrease in the absolute power of slow-wave (0.5–7.5 Hz) in the old (51–85 years, N = 107) group compared to those of the young (18–50 year, N = 108) [32]. Finally, to our knowledge, no study has found statistically significant associations between gamma rhythms in rsEEG signals and aging in healthy groups. Although it was not a resting state, a study focusing on 32 Hz steady-state visual evoked potentials (SSVEPs) showed a decrease in the gamma power of SSVEP with age in a large group of 236 participants [120].

## 4. Nonlinear Neural Dynamics

As an important analytical approach to an EEG time-series signal, nonlinear analysis characterizes the temporal and spatial dynamics in a sequence of EEG data treated as the evolution of an isolated point, called the state vector, over time in phase space. The trajectory of the state vector in a particular coordinated dimension leads to specific temporal and spatial dynamics that can be quantified as a set of nonlinear measurements. A variety of nonlinear techniques have been proposed to extract informative nonlinear dynamics reliably and efficiently by using the underlying specific concepts of dynamical properties, such as complexity, predictability, stability, synchronization, and recurrence, in phase space [21,121]. Regarding the relationship between aging and EEG analysis, various types of nonlinear EEG measures have been proposed to examine the effects of aging on individual measures across the lifespan or to distinguish a particular group across multiple age groups, for example, young vs. older participants. Several types of measurements, such as fractal dimension (FD) [119,122] or entropy-based complexity such as approximation entropy (ApEn) [123], sample entropy (SampEn) [124], and multiscale entropy (MSE) [125], are mainly considered age-related traits (Table 4). Several studies on age-related neural activities in the brain have focused on entropy-based spatial and temporal complexity measures [25,126]. The most well-known theory is that the loss of complexity is associated with aging and disease [127]. According to this hypothesis, physiological aging leads to a general loss of complexity in health dynamics, which impairs the ability to adapt to physiological stress. Based on these findings, complexity measures are good indicators for the assessment of age-related pathological conditions [25].

### 4.1. Fractal Dimension

Theoretically, the fractal dimension refers to the minimum number of fractal coordinates required to organize the EEG signals of the time series into a sequence of state vectors in phase space [128,129,130]. It indicates the spatial complexity in a nonlinear dynamic system; therefore, a higher FD value indicates a higher complexity in the corresponding EEG time-series signals [128]. Two interesting studies on rsEEG signals have reported that the FD value has an inverse U-shaped relationship with the lifespan [119,122]. This means that it increases slightly in adulthood and then decreases significantly with increasing age. These studies examined the value of homologous areas of interhemispheric symmetry (HArS), which are higher when the values of FD in the right region are relatively higher than in the corresponding left region and showed greater asymmetry in older individuals. Although there are differences in the most significant frontal [119] and parietal [122] regions, the patterns of HArS are the same as those of left-higher-than-right FDs in older participants. FD has also been used as an informative feature in ML-based models to predict age from rsEEG signals [53]. In this study, the authors attempted to develop the best models by comparing the performance of several ML-based classifiers with five sets of EEG features computed from spectral, functional, and nonlinear analyses. The FD corresponds to important nonlinear features for age prediction [53].

**Table 4:** Review of age-related nonlinear measures of resting-state EEG signals.

Measures	Study	Main Results	Subjects (Age Range (yr), Numbers (N))	Recording Condition
Fractal dimension	Zappasodi et al. [119]	fractal dimension: increase (<20 yr) decrease (>50 yr)	young (16–25 yr, N = 10) old (25–66 yr, N = 14) older (66–86 yr, N = 16)	REO
	Smits et al. [122]	fractal dimension: inverse U-shape with aging	healthy control (20–89 yr, N = 41)	REC
Entropy	Hogan et al. [124]	sample entropy: young > old	young (21.7 ± 3.1 yr, N = 20) old (73.6 ± 4.1 yr, N = 17) old declined (73.3 ± 4.7 yr, N = 18)	REO REC
	Alu et al. [123]	approximate entropy: young < old	young (24.7 ± 0.5 yr, N = 36) old (70.1 ± 0.9 yr, N = 32)	–
Multiscale entropy	Takahashi et al. [125]	multiscale entropy: young > old	young (29.2 ± 3.8 yr, N = 13) old (64.5 ± 4.2 yr, N = 15)	REC
	McIntosh et al. [131]	sample entropy: young > old	young (22 ± 3 yr, N = 16) middle-age (45 ± 6 yr, N = 16) old (66 ± 6 yr, N = 16)	auditory, visual stimulus
	Waschke et al. [75]	weighted-permutation entropy neural irregularity: increase neural variability: decrease	19–74 yr (N = 19)	auditory stimulus

### 4.2. Entropy-Based Complexity

ApEn, proposed by Pincus [132], is used to quantify the degree of complexity within a time-series signal by measuring the regularity of a dynamic system coordinated by the corresponding signal. Compared to other complexity measures, such as the correlation dimension or the Lyapunov exponent, ApEn has the advantage of robustness, as it can measure more noise or smaller sample sizes by accepting a lower resolution in phase space [25]. However, ApEn tends to overestimate the level of regularity and give inconsistent results. To compensate for these drawbacks, SampEn was proposed by Richman and Moorman [133]. Both ApEn and SampEn are based on the same principle of determining the regularity of a time-series signal based on the frequencies of similar patterns in phase space. Regarding the entropy-based complexity measures with aging, one EEG study examined the significant difference in sample entropy across three groups of participants, young (21.7 ± 3.1 years, N = 20), older control (73.6 ± 4.1 years, N = 17), and older (73.3 ± 4.7 years, N = 18) adults, during four types of EEG recording sessions, namely REC, REO, memory encoding, and memory recognition [124]. Young adults showed a pronounced hemispheric asymmetry on the SampEn, with a higher SampEn in the right temporal and left parietal regions. When looking at the characteristics of EEG-complexity asymmetry in the temporal region, the statistical difference between the right and left areas was significant in the young group and borderline significant in the older group [124]. Another recent study investigated the effect of aging on ApEn in the rsEEG signal acquired from two age groups, young (24.7 ± 0.5 years, N = 36) and old (70.1 ± 0.9 years, N = 32) adults. This study showed that a higher ApEn (higher complexity) was observed in the central, parietal, and occipital areas in the old group, indicating less cerebral connectivity in aging processes [123]. In contrast to the loss of complexity theory, both entropy-based complexity studies on aging have shown that a higher complexity is observed in the younger group than in the older group, although there are some corresponding areas.

### 4.3. Multiscale Entropy

Like ApEn and SampEn, MSE was also developed to estimate the complexity of time-series signals. However, unlike entropy-based complexity measures, MSE is fundamentally based on multiple time scales in a signal, which can be expressed as fine- or coarse-grained sequences [134,135]. The use of multiple coarse-grained sequences enables the detection of memory or history effects in time series across multiple time scales. It enables the distinction between noise and meaning complexity and captures long-range temporal correlations in the dynamics [134,135]. Based on information-processing theory, a series of EEG and MEG studies focused on the relationship between resting-state neural dynamics and behavioral outcomes in cognitive tasks and the effects of aging on the properties of dynamic systems. To accomplish this, these studies mainly used rest–task–rest experimental paradigms and employed MSE to measure the complexity or variability in neural signals during pre- and post-rest conditions. Takahashi et al. recruited 28 participants, including 13 young (29.2 ± 3.8 years) and 15 older (64.5 ± 4.2 years) healthy individuals, to perform the intermediate task as a passive response on photic stimulation (PS). The EEG signals recorded before and after PS in the resting state were used to calculate the MSE, and significant changes in the MSE were examined in both age groups. The difference in MSE between the two groups was not statistically significant; however, significantly higher MSE after PS was observed only in young participants. Based on the above evidence, this study supports the hypothesis that a loss of complexity and reduced functional response is observed with increasing age [125]. McIntosh et al. obtained EEG and MEG data from individual cognitive tasks. In the EEG session, individuals belonging to three age groups, young (22 ± 3 years, N = 16), middle aged (45 ± 6 years, N = 16), and old (66 ± 6 years, N = 16), participated in visual perceptual matching and delayed-match-to-sample tasks. While the EEG signals were not recorded in a pure resting state that were built into 0.5–2.0 s epochs with 0.5 prestimulus, the neural complexity measured by the MSE in those signals revealed two interesting findings related to aging: entropy increased at fine scales and lowered at coarse scales with increasing age. The authors interpreted this as evidence that aging leads to a neural shift from long-range connections to local processing [131]. As an extension of the McIntosh et al. study, Wang et al. intensively investigated the complexity of 2.5 s EEG signals with eyes open during the resting state before and after the task in groups of young, middle-aged, and older subjects [17]. The neural complexity in the EEG signal was calculated using MSE and could be divided into two temporal scales: fine (2–16 ms) or coarse (40–50 ms). Partial least squares (PLS) analysis assessed the trend or difference in the two temporal scales of MSE with aging groups and revealed age-related changes in resting-state dynamics across the MSE. Consistent with the findings of McIntosh et al., young participants had less MSE on the fine scale and more MSE on the coarse scale in the parietal cortex and posterior cingulate compared to the older adult group. This supports the hypothesis that aging leads to a shift from the distributed neural population to local neural processing in posterior areas [17]. Finally, one study on neural noise in the neocortex associated with information processing used weighted-permutation entropy (WPE) to measure neural irregularity (complexity and variability) in EEG signals during prestimulus durations. It investigated the effect of complexity in ongoing EEG signals on sensory encoding and perceptual decisions [75]. By assessing the intraclass correlation coefficients of the pre-stimulus WPEs of healthy participants (19–74 years, N = 19), this study found that the participants’ age correlated positively with mean WPE, with EEG variability decreasing from trial to trial and EEG frequency spectra becoming flatter. It was emphasized that the measurement of neural activity during ongoing activity plays an important role in the prediction of sensory and perceptual decisions (neural state) and the changes of these nonlinear dynamics in aging (neural traits) [75].

## 5. Spatial Topography

### 5.1. Brain Connectivity

Brain connectivity analysis characterizes the functional (unidirectional) and effective (bidirectional) connections in the brain using multidimensional neurophysiological data, such as fMRI and EEG signals [20,26]. For decades, numerous studies on brain connectivity have investigated the specific characteristics of pathological and psychological disorders and various cognitive states in the brain by evaluating meaningful measures of brain properties based on graph theory [136,137,138,139,140]. Before presenting the effects of aging on EEG connectivity during the resting state, we address overall resting-state fMRI connectivity in the context of aging. Indeed, fMRI studies have focused on examining BOLD signals recorded in the resting state to reveal changes in certain spatial and functional features with age. There are two well-known hypotheses related to aging: the hemispheric asymmetry reduction in older adults (HAROLD) [141] and the posterior–anterior shift in aging (PASA) [104]. In the HAROLD model, older adults showed a significant reduction in functional hemispheric lateralization (more homogenous and more bilateral) in the prefrontal cortex (PFC) during task performance compared to younger people. In the PASA model, older adults tend to show less activation of posterior regions and increased activation of anterior regions. This suggests that older people have a frontal over-engagement and a decline in the functional integrity of the posterior regions. The HAROLD model is strongly associated with episodic memory encoding and retrieval, working memory, perception, and inhibitory control [141]. The PASA models are not restricted to a specific cognitive function and can potentially affect activity at rest [142]. With regard to the DMN and its relationship to other networks, a decrease in large-scale connections related to anterior–posterior connectivity was observed in the DMN with increasing age, which can be attributed to disruptions in white-matter integrity [143]. In addition, reduced segregation in the sensorimotor system has been associated with aging. Aging has been reported to lead to a decrease in intra-system correlations and an increase in inter-system correlations [144]. In contrast to the spatial and anatomical structures of the BOLD signals, the EEG connectivity analysis is based on the temporal and spectral dynamics of the multidimensional signals. For simplicity, we divided a study of EEG connectivity related to aging into spectral and nonspectral connectivity, which were examined within a specific frequency band before the connectivity measurements were made (Table 5). For example, the analysis of phase synchrony and its different variants, namely, phase-lag index (PLI), mean phase coherence (MPC), and cross-frequency coupling (CFC), were restricted to specific frequency bands of interest, as these analyses are mathematically based on the degree of phase synchronization within or between predefined narrow frequency ranges across multiple EEG signals. Derived from the cross-spectral density matrix resulting from the multivariate autoregression (MVAR) model across multiple EEG signals, other methods of spectral connectivity, such as spectral coherence, spectral Granger causality, and partial directed coherence, have been widely used to characterize functional and effective connectivity in the brain.

Previous studies have addressed the relationship of aging to alpha and beta rhythms in the context of spectral connectivity in aging humans. Knyazev et al. investigated the differences in the number of important hubs between younger (21.8 ± 3.0 years, N = 76) and older (64.3 ± 6.7 years, N = 70) participants. To this end, they performed a phase synchronization analysis of REO and REC EEG signals to individually construct five types of functional connectivity within the delta, theta, alpha, beta, and gamma rhythms and then identified a group of important hubs in the brain. These experiments showed a significant decrease in global connectivity and fewer hubs in the posterior alpha and beta rhythms with increasing age but not in the delta, theta, and gamma rhythms [31]. Moezzi et al. investigated the properties of five connectivity networks corresponding to the delta, theta, alpha, beta, and gamma rhythms. They used them as input vectors for a support vector machine (SVM)-based classifier to classify binary age groups as younger (24.3 ± 6.1 years, N = 22) and older (71 ± 6 years, N = 22) adults with 93% accuracy in age group classification [145]. In contrast to the findings from the Knayzev et al. study [31], Moezzi et al. found that, with age, the global connections in the beta rhythm increased, but those in the delta, theta, alpha, and gamma rhythms decreased. The most significant decrease in the weight of connections with increasing age was observed in the posterior regions of the alpha rhythm [145]. In addition, a neurodevelopmental study found a significant difference in corticocortical and thalamocortical connectivity between the delta, theta, alpha, and beta rhythms. Using REO and REC signals from two different age groups of school-aged children (10.1 ± 1.3 years, N = 17) and adults (25.1 ± 3.8 years, N = 17), the authors compared the differences in directed source-level brain networks constructed using dynamic imaging of coherent sources and renormalized partial directed coherence methods [34]. They observed two outcomes. In adults, corticocortical information flow was observed in all rhythms from parietal to frontal sources, but in children, it was observed in the opposite direction (from frontal to parietal sources). In addition, there was unidirectional thalamocortical connectivity (from the thalamus to other cortical regions) with the alpha and beta rhythms in adults; however, this connectivity was bidirectional in children [34]. Another study reported that the alpha rhythm is strongly affected by aging compared to other rhythms [99]. Scally et al. reported that not only is there a significantly slowed individual alpha peak frequency but also reduced global power and connectivity in the upper alpha band (10–12 Hz) by comparing different measures of phase synchronization of REC-EEG signals in the two age groups of older (69.8 ± 4.9 years, N = 32) and young adults (20.3 ± 2.1 years, N = 37) [99].

In the context of nonspectral connectivity in aging, Petti et al. constructed weighted and directed connectivity in REO-EEG signals using partial directed coherence based on a general linear Kalman filter acquired from healthy participants (20–63 years, N = 71) and individually computed five measures to characterize the corresponding networks: node strength, characteristic path length, clustering coefficient, (global and local) efficiency, and weight [146]. Through a correlation analysis of these measures across the lifespan, they showed that weight, clustering, and local and global efficiency decreased linearly, but path length increased linearly with aging. They reported that the brain network became more random with increasing age. Since the accuracy on additional binary classification tasks was greater than 80% in the young (23.8 ± 1.1 years, N = 20) and middle age groups (46.1 ± 5.3 years, N = 20), they also established the usefulness of these network measures in manifesting the effects of aging [146]. Consistent with the findings of Petty et al., a more recent study also supports the randomly organized network in aging. Javaid et al. examined the significant differences in network-efficiency matrices computed from REO and REC EEG signals between middle-aged (50.5 ± 5.8 years, N = 20) and older participants (71.0 ± 5.5 years, N = 20) and found that aging produces a more random brain network by demonstrating a significant decrease in node strength, clustering coefficients, and local and global efficiency in the older adult group [147]. Modularity is one of the most important network measures for aging. Modularity measures the degree to which a network can be divided into distinct and relatively unrelated modules, called groups or clusters. Higher modularity scores indicate a greater division of the network into separate modules, which generally indicates a well-organized or well-segregated network. In a recent study, the difference in the modularity index between younger (25–35 years, N = 30) and older (60–80 years, N = 30) people was examined using the LEMON database [142]. The REO and REC EEG signals were localized using the exact low-resolution brain electromagnetic tomography (eLORETA) method, and two types of functional connectivity across sources were constructed using a cross-correlation time scale and mutual information (MI). When comparing the modularity index values of these constructed networks, the authors found that older people exhibited significantly lower modularity than young adults. In other words, older people exhibited less-segregated network structures in which different and unrelated areas were more strongly interconnected. This result is consistent with the decreasing segregation of the brain network with increasing age.

**Table 5:** Review of age-related spatial measures of resting-state EEG signals.

Measures	Study	Main Results	Subjects (Age Range (yr), Numbers (N))	Eyes Condition
Brain connectivity	Michels et al. [34]	RPDC (adults): parieto-occipital → fronto-central	children (10.1 ± 1.3 yr, N = 17) adults (25.1 ± 3.8 yr, N = 17)	REO REC
	Knyazev et al. [31]	Lagged-phase synchronization number of hubs: young > old (more random and less connected)	young (18–35 yr, N = 76) old (51–80 yr, N = 70)	REO REC
	Petti et al. [146]	Partial directed coherence efficiencies, path length, clustering, & global strength: decrease	20–63 yr (N = 71)	REC
	Scally et al. [99]	PLI, WPLI (upper alpha): young > old	young (20.30 ± 2.06 yr, N = 37) old (69.75 ± 4.91 yr, N = 32)	REC
	Moezzi et al. [145]	imaginary coherence alpha: young > old beta: young < old	young (19–37 yr. N = 22) old (63–85 yr, N = 22)	REO
	Javaid et al. [147]	global and local efficiency: decrease clustering coefficient: decrease node strength: decrease	middle-age (41–60 yr, N = 20) elderly (61–84 yr, N = 20)	REO REC
	Perinelli et al. [142]	intra-area connectivity: parietal, temporal: young < old frontal: young > old	young (25–35 yr, N = 30) old (60–80 yr, N = 30) from LEMONDB	REO REC
EEG microstates	Koenig et al. [148]	asymmetric: decrease symmetric: increase	6–80 yr (N = 496)	REC
	Tomescu et al. [149]	gender effect (only in old): microstate C and D	6–87 yr (N = 179)	REC
	Zanesco et al. [45]	microstate A,B (GEV): young < old microstate C,E (GEV): young > old mean duration (all): young < old	young (25–35 yr, N = 153) old (59–77 yr, N = 74) from LEMONDB	REC

### 5.2. EEG Microstates

For whole-brain network properties in multiple EEG signals, an EEG microstate analysis is crucial to detect temporal changes in a series of global patterns of scalp potential topographies. Based on the demonstration of the existence of a quasi-stable spatial distribution system for several milliseconds, all time-series EEG signals can be interpreted in terms of different patterns of spatial distribution called EEG microstates [150,151]. In general, EEG microstates were initially constructed using the global field power as the root mean square of the squared potential differences at all electrodes divided by the number of electrodes and identified as a fixed number of microstates by a k-means clustering analysis. For decades, there have been some questions regarding the possible number of EEG microstates required to optimally represent the majority of EEG signals with only a few topographies. Numerous studies have reported that only four clustering maps can explain the proportion of global variance of 64–84% [152]. These four dominant microstates were labeled microstates A, B, C, and D [19]. The spatial properties of these four microstates were defined as the right-frontal left-posterior (microstate A), left-frontal right-posterior (microstate B), midline frontal–occipital (microstate C), and midline frontal (microstate D) topographies. The characteristics of these microstates were represented by various measures, such as average duration, frequency of occurrence, coverage, global variance, and the transition probability of a particular microstate to others.

Unsurprisingly, previous rsEEG studies focused on the relationship between EEG microstate measurements and aging (Table 5). Koenig et al. extracted the four microstate class topographies in the REC EEG signals of recruited participants of all ages (6–80 years, N = 496) [148]. They demonstrated the significant interactions of microstates with age and developmental stage by eliciting that the asymmetric microstates (belonging to microstates A and B) diminished while symmetric microstates (belonging to microstates C and D) increased with age. Moreover, the changes in these patterns differentiated the four distinct developmental stages: childhood (<12 years), early adolescence (12–16 years), late adolescence (>16 and <21 years), and adulthood (>21 years) [148]. Another EEG microstate study by Tomescu et al. reported significant differences in the dynamics of these four EEG microstates in relation to sex and age by comparing several parameters for each microstate in the REC-EEG signals of participants of all age groups (6–87 years, N = 179) [149]. Not EEG microstates A and B, but EEG microstates C and D have the primary effect and interaction with sex and age accompanied by developmental stages [149]. A recent study explored the meaningful correlates of temporal dynamics of rsEEG microstates using the LEMON database [45]. They selected five representative EEG microstates of global field power using k-means clustering and extracted three parameters: the global explained variance, mean microstate duration, and frequency of occurrence of each microstate. Regarding the main effect of age, older participants had a greater globally explained variance (GEV) for microstates A and B and a lower GEV for microstates C and E. In addition, this study showed that older adults tended to have a long duration and low occurrence of microstates, which is consistent with the results of the two studies mentioned above [148,149].

## 6. State-of-the-Art Signal Processing and AI Models

### 6.1. Riemannian Manifold

Mathematically, the overall procedure of EEG analysis, from obtaining raw signals to finally deciding on specific goals, is deeply associated with a series of linear operations of the matrix [153]. For example, the raw EEG signals recorded at the scalp sites are linked to the EEG sources in the brain by a lead-field matrix, which is calculated linearly by solving the inverse problem. A variety of spatial filters, which are useful tools to improve the signal-to-noise ratio during EEG preprocessing, are theoretically based on linear operations between time-series signals and trained weights of spatial filters. Several linear classifiers, such as the Naive Bayes classifier, linear discriminant analysis, and SVM, have been used extensively for BCI applications. From the perspective of linear operations, some EEG studies have focused on the advantage of manipulating informative EEG features on the Riemann manifold for the classification and detection of empirical data. In particular, the covariance structure of multichannel EEG signals, such as covariance and cross-spectral matrices, has been widely used as one of the most useful EEG features that enable the visualization of informative spectral features in single EEG signals and the construction of brain networks in specific frequency bands derived from functional or effective connectivity across multichannel EEG signals. Due to the property of symmetric positive definition in covariance matrices, it can be used as a set of samples, features, and vectors on a Riemannian manifold satisfied with some properties of distance (non-negative, symmetric, and triangular inequality) in the metric distance space. Under these conditions, all information on the Riemannian manifold at a given point can be invariantly projected onto the tangent space (Euclidean space) using a logarithmic map [154,155]. In contrast to statistical source localization or unsupervised spatial filtering approaches that explicitly aim to generate or infer source-level neural activity in the brain from sensor-level electrical activity on the scalp, manipulation of covariance features in the Riemann manifold is directly linked with the linear operation associated with regression, classification models, and signal processing [9].

For example, Riemannian manifolds are of interest in EEG-based BCI studies [154,155,156]. Barachant et al. developed a Riemannian-based kernel algorithm to classify binary or multiclass motor imagery trials. In this Riemannian-based kernel, half vectorizations of the covariance matrices obtained from the EEG signals of the MI trials were projected onto the tangent space at a given reference point as the geometrical mean on the Riemannian manifold. A linear SVM classifier used these EEG features processed on the kernel and performed better than the common spatial pattern method, the most popular spatial filtering method in BCI applications [154,156]. Recently, two interesting EEG studies have used EEG processing on the Riemannian manifold to predict a person’s age [9,50] (Table 6). First, Sabbagh et al. reported the superior performance of the Riemannian manifold for predicting the age of a participant by comparing three types of regression models (upper, Riemannian, and source power comodulation) in combination with the optimal tuning of multiple hyperparameters and functional options. To do this, a large-scale EEG database, which is named the TUH database and was introduced in Section 2.1. in this review, was used. The least mean absolute error (MAE) of age prediction in the Riemann regression models was consistent with the results of another large-scale MEG database [9]. Second, an international EEG research consortium studies the age-dependent developmental trajectories of the brains of healthy and diseased individuals [50]. To achieve this goal, they focus on the proper definition of descriptive parameters (DPs) that characterize the information on anatomical and physiological features in the brain and investigate the effect of age on these DPs and age prediction using the DPs. As one of the best examples of age-dependent DPs, the cross-spectral matrix was primarily adopted due to the advantage of EEG manipulation on the Riemannian manifold. In short, a cross-spectral matrix can be defined as the product of spectral transformations using a multi-tapper approach or Fourier and wavelet transforms of multiple time-series signals so that it can be used to identify the spectral relationship between them. As a form of cross-covariance in the frequency domain, the cross-spectral matrix has a symmetric and positive-definite Hermitian property that enables the manipulation of the Riemannian manifold. In this study, a new DP, called the HarMNqEEG norm, was defined by projecting these cross-spectral matrices onto the Riemannian manifold in combination with the z-transform for age-independent brain-development deviations. They created a large-scale EEG database with data from nine countries and extensively studied the relationship between EEG norms and aging.

### 6.2. CNN/RNN Models

To develop reliable models for predicting a person’s age from rsEEG, recent studies have actively attempted to adopt state-of-the-art ML or DL algorithms to reveal the statistical relationship of lifespan aging with a bundle of individual EEG features and distinguishing specific age groups (Table 6). In line with other AI research fields, previous regression models and classifiers for aging in EEG signals were originally based on various decision trees or artificial neural networks (ANN). Several famous tree-based models can be learned using ensemble methods, such as bagging, boosting, and stacking. These include random forest and gradient boosting algorithms and their variants. In ANN, the convolution neural network (CNN) and recurrent neural network (RNN) underlie several previous DL-based models related to the aging of EEG signals. Van Leeuwen et al. adopted the architecture of CNN from EEGNet, which is one of the famous CNN-based classifiers for BCI application [157], to predict the stage of age and sleep [158]. The 1 min 18-channel EEG signals, which are sourced from a database of 8522 EEG signals of patients aged 18–85 years, were passed through the CNN-based classifiers and resulted in the classification of three age groups (18–29, 30–39, and 40–49 years) with an area under the receiver operating characteristic (AUC) performance of 0.924 [158]. Using the Tulsa-100 database, Al Zoubi et al. developed the brain age gap estimation (BrainAGE) model, which consists of a general linear model to combine the weights from a set of five different regression algorithms to estimate age from EEG signals. This model used a set of five EEG features as the input and resulted in an age-estimation performance with a correlation of 0.6 across the lifespan [53]. Age predictions based on CNN models have also become popular. In a recent study, age prediction was performed on the TD-Brain EEG database with 1346 EEG sessions (1274 participants with a mean age of 38.67 ± 19.21 years) by using CNN-based models with 5 s 26-channel EEG signals during both REO and REC. The authors reported that the proposed model achieved an MAE of 5.96 years between the actual and predicted age and emphasized the central role of the frontocentral area in age prediction [159].

In the RNN-based model, a recent EEG study intensively employed long short-term memory (LSTM)-based approaches for age prediction [160]. As an RNN variant, the LSTM method avoids the main drawback of RNN architecture, known as the vanishing gradient problem, which results in insufficient weight updates. Kaushik et al. (2019) employed a hybrid deep bidirectional LSTM (BLSTM)-LSTM network to classify six age groups from 6–55 years [160]. The REC-EEG signals recorded by the smart EEG device (Emotive Epoc) were first filtered within the five spectral bands using the discrete wavelet transform (DTW), and these filtered signals were used in the stacked deep BLSTM-LSTM model. Among these, the beta rhythm (12–30 Hz) resulted in the highest classification accuracy of 93.69% in the six age groups. In contrast to the RNN-based model, the same emotive EEG dataset was used in the study by Kaushik et al. in other age-prediction studies [161]. Like the Kaushik et al. study, Kaur et al. also used the same six age groups and performed the same DWT to filter the five EEG rhythms. Compared to the SVM and ANN classifiers, the random forest classifier performed best in classifying the six age groups with an accuracy of 88.33%. Among the five bands, the beta rhythm was the most informative for age prediction. Based on the evidence of the usefulness of the beta rhythm in the above two studies, a more recent study also focused on the beta rhythm to perform regression analysis and classify the six different age groups, which consisted of patients aged ≥30 years, by re-sampling for a more balanced dataset from a total of 564 participants of TUAB. Compared to several RNN variants, they showed that BLSTM models with the beta rhythm as an input performed best in age classification [7]. Unlike other studies that have suggested spectral properties associated with age, these three LSTM studies have consistently examined the feasibility of beta rhythm associated with age.

### 6.3. Self-Supervised Learning Model

From the perspective of model training in ML or DL approaches, most of the age-related regression or classification models presented in this review belong to supervised learning. In supervised learning, the procedure of model training for high performance, in which the optimal weights of all corresponding hidden layers in the network are determined by iteratively updating these weights from the initial random parameters, requires a large set of labeled training data. However, creating a reliable training dataset that is labeled or annotated by experts is expensive and time consuming. Moreover, since model training from initial random weights in supervised learning cannot guarantee optimal weights for high performance, alternative training methods that do not require labeled datasets have been proposed to reduce the shortcomings of supervised learning. As an unsupervised learning method, self-supervised learning (SSL) models have gained acceptance in various practices and research areas of DL models. Recently, sophisticated studies have been conducted in EEG research to develop regression and classification models for various purposes [162,163,164].

Recent EEG studies have adopted the SSL approach to perform two individual classification tasks, which correspond to the discrimination of sleep stages (five classifications) and the detection of normal EEG (two classifications) [43] (Table 6). To achieve this, the authors trained two types of CNN-based classifiers by using the SSL approach, which consists of two different tasks: pretext and downstream. A pretext task is an auxiliary task used to generate surrogate signals using unlabeled data. It is used to pre-train a deep neural network using a large dataset without much annotated data. The network learns to solve these pretext tasks, allowing it to learn meaningful representations and features from the data. These learned representations can then be transferred and fine-tuned for downstream tasks for which only a small amount of labeled data is available. Downstream tasks refer to actual tasks or applications to which the learned representations of the pre-trained model are transferred and fine-tuned. After pre-training the model on pre-wall tasks with unlabeled data, the representations learned by the model can be used as a starting point for various supervised or unsupervised tasks where labeled data is limited or expensive to obtain. The main advantage of SSL is that the model can learn useful representations from a large amount of unlabeled data and transfer this knowledge to downstream tasks, which often contain relatively few labeled samples. To pre-train the models, three types of default tasks were performed: relative positioning, temporal shuffling, and contrastive predictive coding to generate a bundle of surrogate datasets to solve artificial problems. The pretrained CNN model based on StagerNet was applied to a specific classifier for five sleep stages in the Physionet EEG database and successfully achieved a maximum performance of 72.3%. Another CNN classifier based on ShallowNet classified abnormal and normal EEG signals in the TUAB database with an accuracy of 79.4% [43]. Extending the study by Banville et al., Wagh et al. proposed EEG classifiers based on the Resnet-18 backbone in SSL for three specific goals [8]. Among them, the binary classification for young (<45 years) versus old (>45 years) age groups was examined using the TUAB and LEMON databases. Pretext tasks consisted of three types of SSL tasks: hemispheric symmetry (HS), behavioral state estimation (BSE), and age-contrastive (AC) tasks. Expressed in individual terms, the HS pretext exploits the properties of hemispheric symmetry across real and augmented datasets. The BSE bias utilizes the relationship between delta and beta powers, and the AC tasks use the contrastive triplet defined by the difference between the young and old age groups. Compared to the combination of pretext tasks and CNN-based models, the Resnet-18 backbone model trained in both the BSE and AC pretext tasks showed the best performance of 0.987 AUC values for binary age classification in the LEMON database. This study is a good example of a state-of-the-art SSL model effectively used for processing EEG signals and age prediction.

**Table 6:** Review of artificial intelligence methods for age estimation by resting-state EEG signals.

Study	Subjects (Age Range (yr), Numbers (N))	Key Methods	Best Results (Classification, Regression)
Sabbagh et al. [9]	TUAB DB (10–95 yr, N = 1385)	Covariance matrix Riemmannain	MAE: 8.21 yr (see the Figure 5 in [9])
Li et al. [50]	1564 EEGs from 9 countries (including CHBMP DB)	HarMNqEEG Riemmannain	-
Van Leeuwen et al. [158]	18–85 yr (N = 8522)	CNN	AUC: 0.924 (classification across 3 age groups)
Al Zoubi et al. [53]	T–1000 DB (mean age: 34.8 yr, N = 468)	5 ML models	MAE: 6.87 ± 0.69 yr RMSE: 8.46 ± 0.59 yr
Engemann et al. [6]	LEMON DB (20–77 yr, N = 227) CHBMP DB (18–68 yr, N = 282) TUAB DB (10–95 yr, N = 1385)	5 approaches	LEMON DB (MAE: 7.75 ± 1.78 yr) CHBMP DB (MAE: 6.48 ± 0.60 yr) TUAB DB (MAE: 7.75 ± 0.56 yr)
Khayretdinova et al. [159]	TD-Brain DB (5–88 yr; N = 1274)	CNN	MAE: 5.96 ± 0.33 yr
Kaur et al. [161]	6–55 yr (N = 60)	Random Forest	Accuracy: 0.883 (classification across 6 age groups)
Kaushik et al. [160]	6–55 yr (N = 60)	Deep BLSTM-LSTM	Accuracy: 0.937 (classification across 6 age groups)
Jusseaume et al. [7]	TUAB DB (2–88 yr, N = 388)	BLSTM	Accuracy: 0.90; MAE: 6.5 yr; RMSE: 9.1 yr (classification across 6 age groups)
Banville et al. [43]	TUAB DB (10–95 yr, N = 1385)	SSL	Not aging prediction classification accuracy: 0.794 (between normal and abnormal EEG)
Wagh et al. [8]	TUAB DB (10–95 yr, N = 2328) LEMON DB (20–77 yr, N = 216)	SSL	AUC: 0.872 (TUAB), 0.987 (LEMON) (classification across young and old)

## 7. Conclusions

Aging significantly mediates a variety of characteristics that are reflected in a set of local or global EEG features based on the linear or nonlinear dynamics of EEG signals. Although the trend of life-course changes in individual EEG features remains controversial, we can summarize the four important age-related characteristics of rsEEG signals as follows (Table 7).

First, aging slows the frequency of the alpha rhythm in EEG signals, which has been closely associated with a decline in cognitive performance and slower information-processing speed [108,165]. The age-related increase in delta and theta rhythms in senile diseases with dementia might be quantitatively measured as the decrease in the median, center frequencies, or individual alpha peak frequency [18,59,99]. Second, aging causes random EEG signals. However, the meaning of the term randomness should be interpreted carefully. In this case, it means the flatness of the 1/f noise in the PSD of a single EEG signal, which is mainly measured as a decline in the aperiodic exponent [76,82]. These patterns consistently show that aging increases background noise and reduces the signal-to-noise ratio, which consequently interferes with the inhibitory control of neural mechanisms [69,166] related to pathological diseases, with declines in cognitive and motor performance [68,71,167]. Third, aging hinders the neural efficiency of brain networks that are constructed using EEG connectivity methods with network dynamics [146,168,169]. Compared with the well-organized brain networks of young healthy adults with good neural differentiation and network segregation, which have a high degree of modularity, older adults have a tendency towards neural inefficiency due to the loss of network segregation with low modularity [31,170,171,172]. This evidence indicates that the network connections corresponding to task-relevant and task-irrelevant regions are gradually ignored with aging, which impairs cognitive performance [170,173]. Finally, aging evokes a compensatory mechanism followed by a deficit in neural structures [174,175,176]. Consistent with neuroimaging studies, the fundamental patterns of the bilaterality (HERA) [141] and frontal engagement (PASA) [104] models in EEG networks have been extensively investigated as important examples of age-dependent spatial patterns related to compensatory mechanisms. In particular, age-related EEG asymmetries have been expressed using spectral power or entropy-based EEG complexity, identifying the significant bilaterality in aging [119,122,141,177].

As described above, most previous studies have focused on investigating the relationship between age-related EEG features with aging to understand the underlying neural mechanisms. Recently, however, these features have attracted considerable interest as critical inputs to ML/DL-based models for age prediction and classification. Thus, the development of reliable descriptive parameters obtained from rsEEG signals measured with different EEG hardware devices from multiple institutions worldwide aims to enable more efficient information transfer and management along with the development of a reliable age-prediction model. In addition, EEG models recently developed to solve various issues (related or unrelated to age) require large-scale and complex structures. As a generative model in the DL domain, the SSL model offers a fundamentally different approach to traditional classification models based on feature engineering and linear or quadratic classifiers. In addition to the development of ML/DL-based models, research is needed on estimating the informative role and importance of individual model features and, conversely, the use of models in identifying links with the neural mechanism of aging. As highlighted above, jumping to conclusions without considering the different features of individual EEG measurements in aging and the age range of interest may lead to erroneous results or misconceptions regarding outcomes. In the relationship between spectral power and aging, different selections using absolute or relative power, as well as different types of normalization transformations, may lead to substantially different or even completely opposite results. In addition, defining specific age groups by simply using common terms such as “young” and “older” leads to a hasty conclusion without accounting for cases where the relationship changes dramatically based on specific age ranges during the period of neurodevelopment. While research on the relationship between aging and rsEEG signals remains unclear, advances are underway in several areas, such as novel algorithms for improved measurement of spectral power that separate periodic and aperiodic components for more robust performance, as well as analytical approaches in EEG signals that reconcile differences between neural activity occurring in sources and EEG signals measured by sensors by processing in manifold dimensions of the feature domain. Given increasing societal interest in aging, EEG research is expected to lead to technological advances.

**Table 7:** Summary of main age-related characteristics and the corresponding relevant evidence.

Age-Related Characteristics	Study	Main Relevant Evidence
Slow rhythm	Stacey et al. [23]	peak frequency: young > old
	Scally et al. [99]	IAPF: young > old
	Donoghue et al. [72]	center frequency: young > old
	Chiang et al. [96]	peak frequency: younger > older
	Cesnaite et al. [59]	IAPF: decrease
Randomness or Regularity	Voytek et al. [68]	1/f slope at visual, parietal, frontal: young > old (flatten)
	Donoghue et al. [72]	exponent, offset at Cz: young > old (flatten)
	Pathania et al. [82]	exponent at frontal, central, parietal: young > old (flatten)
	Cesnaite et al. [59]	1/f slope at fronto-central: decrease (flatten)
	Alu et al. [123]	approximate entropy at central, parietal, occipital: young < old
	Nobukawa et al. [171]	complexity of DPS at frontal alpha: young < old
	Knyazev et al. [31]	number of hubs at posterior: young > old (more random)
	Waschke et al. [75]	neural irregularity: increase; neural variability: decrease
	Hogan et al. [124]	sample entropy: young > old
	Takahashi et al. [125]	multiscale entropy: young > old
	McIntosh et al. [131]	sample entropy: young > old
Neural inefficiency	Petti et al. [146]	efficiencies, path length, clustering, global strength: decrease
	Javaid et al. [147]	global, local efficiency, clustering coefficient, node strength: young > old
	Knyazev et al. [31]	number of hubs at posterior: young > old (less connected)
	Perinelli et al. [142]	modularity: young > old
	Nobukawa et al. [171]	interhemispheric connectivity at frontal alpha: young > old
	Scally et al. [99]	PLI, WPLI (upper alpha): young > old
Spatial alternation	Michels et al. [34]	RPDC: from parieto-occipital to fronto-central
	Moezzi et al. [145]	imaginary coherence: alpha: young > old; beta: young < old
	Perinelli et al. [142]	intra connectivity: frontal: young > old; parietal, temporal: young < old
	Koenig et al. [148]	asymmetric microstates: decrease; symmetric microstates: increase
	Zanesco et al. [45]	mean duration, microstate A,B: young < old; C,E (GEV): young > old

## Figures and Tables

**Table 1:** Review of age-related aperiodic measures of resting-state EEG signals.

Measures	Study	Main Results	Subjects (Age Range (yr), Numbers (N))	Eyes Condition
Aperiodic components	Cellier et al. [78]	exponent, offset: decrease	adolescence (3–24 yr, N = 96)	REO
	Hill et al. [83]	exponent, offset: decrease	early–to–middle childhood (4–12 yr, N = 139)	REO, REC
	Tröndle et al. [57]	slope, intercept: decrease	adolescence (5–22 yr, N = 2529)	REO, REC
	Voyetk et al. [68]	1/f slope: young > old (flatten)	young (20–30 yr, N = 11) old (60–70 yr, N = 13)	*–*
	Donoghue et al. [72]	exponent, offset: young > old (flatten)	young (20–30 yr, N = 17) old (60–70 yr, N = 14)	REO
	Pathania et al. [82]	exponent: young > old (flatten)	young (<35 yr, N = 22) old (>59 yr, N = 24)	REC
	Cesnaite et al. [59]	1/f slope: decrease (flatten)	LIFE–Adult DB (40–79 yr, N = 1703)	REC

**Table 3:** Review of age-related delta and beta rhythms measures of resting-state EEG signals.

Measures	Study	Main Results	Subjects (Age Range (yr), Numbers (N))	Eyes Condition
Beta power	Marciani et al. [106]	relative power: younger < old	young (29.9 ± 10.4 yr, N = 21) old (60.6 ± 5.4 yr, N = 20) older (78.7 ± 7.3 yr, N = 19)	REC
	Zappasodi et al. [119]	power: inverse U-shape with aging	young (16–25 yr, N = 10) old (25–66 yr, N = 14) older (66–86 yr, N = 16)	REO
	Caplan et al. [100]	(BOSC) power: young < old	young (18–30 yr, N = 16) old (60–74 yr, N = 12)	REO REC
	Wang et al. [17]	relative power: young < (middle-age, older)	young (22 ± 3 yr, N = 16) middle-age (45 ± 6 yr, N = 16) older (66 ± 6 yr, N = 16)	REO
	Al Zoubi et al. [53]	relative power: increase	T–1000 DB (mean age: 34.8 yr, N = 468)	REO
	Stacey et al. [23]	power: young < old	young (18–30 yr, N = 31) old (61–90 yr, N = 44)	REO REC
Delta power	Babiloni et al. [108]	power: young > old	young (18–50 yr, N = 108) old (51–85 yr, N = 107)	REC
Slow wave (0.5–7.5 Hz)	Whitford et al. [32]	power: decrease	10–30 yr (N = 138)	REC
